# Editorial: Natural immunomodulators in veterinary medicine

**DOI:** 10.3389/fvets.2024.1461975

**Published:** 2024-07-19

**Authors:** Hetron M. Munang'andu, Dagmar Mudronova, Peter Popelka

**Affiliations:** ^1^Faculty of Biosciences and Aquaculture, Nord University, Bodo, Norway; ^2^Department of Microbiology and Immunology, University of Veterinary Medicine and Pharmacy in Kosice, Košice, Slovakia

**Keywords:** immunomodulators, plant polysaccharrides, immunostimulants, adjuvants probiotics, immunosuppressants, veterinary medicine, herbal medicines

## Introduction

Immunomodulators are substances that optimize the immune system to work more efficiently. Although the immune system inherently produces cells capable of recognizing and eliminating foreign agents, it can become weak needing the help of immunomodulators. As such, immunomodulators can serve as immunostimulants capable of increasing immune responses above basic levels. Immunostimulants are useful for treating clinical conditions like chronic inflammation, infections, and cancers. Immunomodulators can also serve as adjuvants able to enhance antigen uptake to boost vaccine efficacy. They can function as immunosuppressants needed for treating autoimmune diseases and organ transplant rejections. Although natural products from plants, and microorganisms are used to treat diseases in animals their role as immunomodulators has not been widely investigated in Veterinary Medicine like in human medicine. Their mode of action, the ability to potentiate the immune system, and their role as growth promoters in animals have attracted research interests in recent decades. Despite so, there are limited reviews on natural immunomodulators used in Veterinary Medicine. Thus, the current Research Topic aims to provide an overview of the current state of knowledge about the immunomodulating role of substances of natural origin in Veterinary Medicine.

## Chinese Yu-Ping-Feng polysaccharides mixture as immunomodulators in livestock

Chinese herbal medicines induce significant immune modulatory effects, antiviral, antioxidant, anticancer, and growth promotion properties in animals. Chu et al. reviewed the impacts of Yu-Ping-Feng polysaccharides (YPF-P) growth performance and immune functions in different animal species where it is used to treat autoimmune diseases, chronic inflammations, cancer, and viral infections. Their review classified the modulatory role of YPF-P into two major functions that include enhancing growth performance and immune functions. As a feed additive in pigs, it alleviates stress in gestating sows, increases farrowing survival rate and litter size, and increases the average weaning weight of piglets whereas in poultry, it promotes chick growth, and regulates the expression of the sodium/glucose transporter 1 (SGLT1), SGLT2, and SGLT5 genes in the intestinal epithelial cells thereby promoting faster nutrition absorption. In cattle, it increases the fattening rate, increases the digestive activity of enzymes like rumen cellulose, gastric protease, and small intestine pancreatic protease. It also increases daily weight gain and enhances fattened cows' high-grade meat production. Their review (Chu et al.) also reported that in dairy cows, it increases milk production while in goats it improves meat quality, formation of beneficial intestinal tract flora, and increase of fatty acids in muscles. As for immunological responses, they pointed out that YPF-P increases the serum antibody levels and the ratio of CD4+ and CD8+ cells in animals suggesting that it enhances both the humoral and cell-mediated immune responses. In poultry, it was shown to increase antibody levels of birds vaccinated against avian influenza using an inactivated oil emulsion vaccine (1). It has also been shown to enhance the humoral and cell-mediated immune responses in chicken vaccinated against infectious bursal disease (2). In pigs, YPF-P was shown to increase the cytokine response of genes like IL-2, and IFN_γ_, and the population of T-lymphocyte subsets like CD3, CD4, and CD8. Altogether, the review by Chu et al. shows that YPF-P enhances growth and modulates the immune response in animals.

## Chinese herbal medicine mixture as an immunomodulator in fish

Wang et al. evaluated the impacts of the Chinese herbal medicine mixture (CHMM) on the immune response of rainbow trout (*Oncorhynchus mykiss*) infected by infectious hematopoietic necrosis virus (IHNV). Their findings showed significant upregulation of immune genes like *NF-*κ*B, TNF-*α, *IFN-*β, *IL-1*β, *JAK1, HSP70*, and *HSP90* in all CHMMs treated fish. In addition, fish infected by IHNV-fed CHMM showed increased total superoxide dismutase, acid phosphatase, and alkaline phosphatase activities and significantly reduced malondialdehyde content. Thus, this study showed that CHMM enhanced the immune response of rainbow against IHNV by serving as an immunostimulant that boosted the antiviral activity in CHMM-fed fish.

## Probiotics as immunomodulators in vaccinated mice

The intestinal microbiota plays an important role in modulating the immune system. As such, the inclusion of probiotics to improve the intestinal microbiota composition leads to improving the immune system. Jesus et al. compared the immune response of mice immunized with the rabies vaccine (Biosyn Animal Health) plus the probiotic Nuxcell Neo^®^ with mice exposed to the rabies vaccine without the probiotic. Nuxcell Neo^®^ is a probiotic consisting of the bacteria *Lactobacillus casei* and yeast *Saccharomyces cerevisiae* together with several vitamin and mineral elements that include folic acid, nicotinic acid, pantothenic acid, antioxidant additive, arginine, copper, choline, phenylalanine, iron, histidine, inositol, iodine, manganese, potassium, selenium, zinc, and vitamins A, B1, B12, B2, B6, and D3. Jesus et al. observed that mice immunized with the rabies vaccine combined with the probiotic Nuxcell Neo^®^ probiotic had higher antibody responses than mice vaccinated with the rabies vaccine without the probiotic. This study showed that probiotics contribute to increasing antibody responses in vaccinated animals.

## Plant polysaccharides as immunomodulators in poultry

Plant polysaccharides are natural immunomodulators that modulate the adaptive, acquired, and innate immune systems by interacting with leukocytes, monocytes, macrophages, and B- and T-lymphocytes. Zhao et al. reviewed the immunomodulatory effects of different plant polysaccharides that included *Atractylodes macrocephala* Koidz polysaccharides (PAMK), *Astragalus* polysaccharides (ASP), Taishan *Pinus massoniana* pollen polysaccharide (TPPP), and alfalfa polysaccharides used to treat different diseases in poultry because of their immunomodulatory, antitumor, antiinflammation, and hypoglycaemic effects. In their review, Zhao et al. pointed out that several plant polysaccharides modulate the transcription of different inflammatory cytokines like IL-1β, IL-2, IL-6, IL-8, IL-10, IL-17 IFN_γ_, and TNFα in poultry. They also pointed out that different plant polysaccharides modulate the expression of cytokines like TGF-β, IL-6, and IL-5 to alleviate immunosuppression. As for the activation of innate and adaptive immune cells, they promote the maturation of macrophages and dendritic cells that stimulate the proliferation of T and B lymphocytes. In their review, Zhao et al. pointed out that plant polysaccharides like TPPP promote the differentiation and proliferation of CD4+ and CD8+ T lymphocytes by upregulating the expression of MHC-I and II genes leading to the differentiation of immature T-cells into effector, regulatory, cytotoxic, and memory T-cell subsets. They also pointed out that plant polysaccharides increase the expression of IgG, IgM, and IgA in serum. A study done by Zhao et al. (3) showed that PAMK increased the antibody responses in chickens vaccinated against Newcastle disease virus while Zhang et al. (4, 5) showed that adding ASP as an adjuvant for avian infectious bronchitis and H5N1 avian influenza vaccines, increased the antibody levels and the expression of inflammatory cytokines like IL-1β, IL-2, Il-8, and TNFα in a dose-dependent manner in vaccinated birds. Other plant polysaccharides reviewed by Zhao et al. include *Epimedium, Salvia miltiorrhiza, Agaricus blazei* Murill, *Enteromorpha, Glycyrrhiza, Acanthopanax senticosus, Artemisia ordosica, Polygonatum sibiricum, Echinacea purpurea, Platycodon grandiflorum*, and *Paulownia fortunei* polysaccharides also shown to modulate the innate and adaptive immune system in poultry.

## Conclusion

Collectively, this Research Topic reveals how scientific research has been used to unravel the functional mechanisms of substances of natural origin as immunomodulators. We hope this Research Topic will stimulate and deepen our knowledge of immunomodulators from substances of natural origin in Veterinary Medicine.

## Author contributions

HM: Conceptualization, Supervision, Validation, Writing – original draft, Writing – review & editing. DM: Conceptualization, Supervision, Validation, Writing – review & editing. PP: Conceptualization, Supervision, Validation, Writing – review & editing.
